# Remittent Fever—Its Prompt Arrest and Effective Treatment

**Published:** 1887-05

**Authors:** John T. Jones

**Affiliations:** New Orleans, La.


### REMITTENT FEVER—ITS PROMPT ARREST AND EFFECTIVE TREATMENT.

---

BY JOHN T. JONES, M. D., NEW ORLEANS, LA.

---

(*Concluded.*)

I do not wish, however, to be understood as claiming that the use of quinine can be relied on to check the course of the fever in every instance. I have myself met with cases in which its employment seemed to be strongly contra-indicated and some which proved intractable to its influence, but these exceptions could always be traced to some recognizable causes, such as a congested or inflamed condition of some part or organs, etc. Dr. Ronald Martin says of it: " A disease so varying in its nature, so general and complicated in its influence on the system, cannot be justly treated by any one remedy." Even when quinine is our main reliance, other symptomatic and preliminary treatment is nearly always required.

Should there be much tenderness of the epigastrium or any nausea and vomiting at the time of or after the administration of the quinine, the application of a mustard poultice, or even a fly blister for a short time, to the region of the stomach will be quickly efficacious, and I have seen the medicine in large doses thus caused to be quietly tolerated when it had been vomited previously in smaller amounts, or when it seemed that not even the blandest substance would be retained.

To stimulate the liver, quiet the stomach, cause a drain from the portal circulation and empty the bowels, in cases seeming to require such treatment, and when there is a sufficient space of time before the proper period for the administration of the quinine, the following prescription, followed in four hours by a brisk saline purge, will be found of use:

℞.—Hydrarg chlorid. mitis . . . . . gr. j.
Hydrarg bichloride . . . . . . gr. $\frac{1}{20}$
M.
S.—Take at once on an empty stomach.

If it is not desired to stimulate the liver the purge may be given alone.

Digitalis in small doses I have thought to yield happy results by its action on the heart when the circulation is feeble and as a diaphoretic and diuretic when the skin is harsh and dry, or when the secretion from the kidneys is scanty and high colored. The practice of sponging the whole surface of the body with tepid water which is allowed to dry off is an effective method of reducing high temperature. Warm pediluvia to equalize the circulation when the feet and hands are cold, or when there is evidence of congestion of the intra-cranial, intra-spinal or other structures are useful though simple measures.

In cases of much encaphalic disorder as a result of congestion or inflammation within the head, the application to it of powdered ice in bags till the pulse begins to become depressed, especially in connection with the use of warm pediluvia at the same time will be attended with very powerful effects. It ought, however, to be resorted to with care and discrimination and be at once discontinued as soon as the pulse begins to flag.

Next to quinine I look upon the application of blisters to the feet during the time of the remission as useful in checking the course of the fever. We may suppose them to act in a manner somewhat similar to the cinchona salt by producing a nervous stimulant impression and as derivatives. It is in cases where quinine cannot be given, or having failed of its usual decisive effect, and the fever being still kept up by the existence of congestion or inflammation of some part or organ along with a deficiency of the circulation in the extremities that resort to the vesicants is more particularly indicated.

Of the beneficial effect of this vesicant treatment, I believe as good an illustration will be furnished, as one example can be supposed to do, by the case of the Geordam children, in Plaquemine parish in 1880, which at that time attracted such wide-spread attention and newspaper comment on account of the then considered equivocal nature of the fever and its malignant fatality with them, five having died out of seven. I had sole charge of the latter part of the treatment of the last three of these children taken sick, two of whom recovered. These two, a boy and a girl, aged respectively about nine and eleven years, had had the disease some five or six days at the time of my taking charge.

The fever was then distinctly of the typhoid type, and the symptoms in both were almost identical. The pulses were fast, thready and feeble; the tongues were dry, coated, brownish-colored and red at the tip and edges, and the teeth and lips were covered with black sordes. There was heat of head with coldness of the extremities, and the pupils were contracted. The condition of their minds seemed languid and stupid, and in one case slight delirium was occasionally present. There was much tenderness to pressure over the epigastrium, and scarcely anything, either food or medicine, would be retained. One of them had some bleedings from the nose, and the other passed some red fluid blood through the bowels. The only symptoms of any abatement that could be detected were a slight moisture of the skin, a tendency of the tongue to clean and become more moist, and a general brightness of the children's whole appearance. When the course of the fever had nearly ceased, the urine collected in the bladder in such large quantities and so rapidly as to require its artificial removal. On first seeing them I applied blisters pretty freely to the calves of their legs, fore-arms and epigastriums at the time that the abatement above described appeared, changing their position from day to day, and, when possible, leaving them long enough to redden the skin, but not to vesicate. The rapidity with which the blistered surface over the epigastrium healed and dried seemed to indicate the existence of some strong counter-derivative influence within. After the second or third day small doses of the solution of the bisulphate of quinia was cautiously tried by administration in the morning with seemingly good results.

The head symptoms were combatted by the ice-caps in connection with warm pediluvia, as before described, repeated when the symptoms seemed most to indicate them. Digitalis in doses sufficient to keep the pulse filled was given to overcome the feebleness of the circulation and for its action on the cutaneous and renal secretory functions. As supporting measures, toddy and beef tea were administered. Under this treatment, though the course of the disease was not at once checked, the violence of some of the symptoms was abated, the gravity of the complications lessened, the fever diminished and the strength supported till convalescence became gradually established, about the fifth or sixth day after my undertaking treatment, and the tenth or twelfth of the disease.

Nervous stimulants, the preparations of camphor, valerian, etc., will find a useful application in conditions of nervous depression, as in the form of low muttering delirium, and especially in the asthenic type of the fever. If there happen to be a condition of cerebral excitation, bromide of potash will often yield happy results by its calmative influence, as well also by its seeming tendency to lessen the determination of blood to the head; but its depressing action on the heart ought always to be counteracted by administering along with it a corresponding dose of digitalis. Chloral being still more of a depressant than the bromide, I have feared to try it, and the philosophy of its employment does not seem well sustained in a disease of such severity, whose febrile manifestations are supposed to be directly brought about by a condition exactly similar to that it produces, viz., central nerve depression. In but a few instances have I had occasion to resort to any other remedies besides these mentioned. Opium in large doses has been recommended by some high authorities for the purpose of causing necessary sleep, but I have never yet given it a fair trial.

Only one thing more in connection with the treatment of this fever do I beg leave to mention here, viz., the necessity of supporting measures. Remembering its great liability when running any protracted course to change from the sthenic to the asthenic type, the thorough prostration which usually accompanies it and the constant tendency to the occurrence of hemorrhage, especially as the result of sudden excitement or general systemic disturbance, the necessity will be easily seen of supporting our patients well with such measures as beef tea and toddy, milk with lime water, milk punch, etc., and keeping him in a quiet, well-ventilated room, without allowing him to be unduly excited or uselessly disturbed, taken in and out of bed, etc., on the most trifling occasions, and the wisdom of their employment will be quickly recognized by a short experience of their use.

The physician who is careful about such matters will save many lives that otherwise would be sacrificed.

